# Branch Duct – IPMN and PanIN, in IgG4‐Autoimmune pancreatitis: A case report

**DOI:** 10.1002/ccr3.2641

**Published:** 2020-09-10

**Authors:** Gianni Lazzarin, Lucia Romano, Gino Coletti, Alessandra Di Sibio, Vincenzo Vicentini, Mohamad W. Alid Fatayer, Mario Schietroma, Beatrice Pessia, Matteo Leone, Francesco Carlei, Antonio Giuliani

**Affiliations:** ^1^ Department of General Surgery Department of Biotechnological and Applied Clinical Sciences University of L'Aquila L'Aquila Italy; ^2^ UOC Anatomia Patologica ASL1 Abruzzo Ospedale San Salvatore L’aquila Italy; ^3^ Department of Radiology ASL1 Abruzzo Ospedale San Salvatore L’aquila Italy

**Keywords:** IgG4 autoimmune pancreatitis, pancreatic branch‐duct intraductal papillary mucinous neoplasm, pancreatic intraepithelial neoplasia, pancreatic worrisome features

## Abstract

The presence of pancreatic lesions in patients with autoimmune pancreatitis requires histological diagnosis (percutaneous or endoscopic biopsy) to exclude malignancy. A nonspecific histology after endoscopic or percutaneous biopsy of a pancreatic lesion may require surgical excision and definite histology.

## Graphical abstract

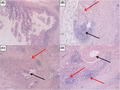

## 1 INTRODUCTION

The simultaneous presence of a pancreatic branch‐duct intraductal papillary mucinous neoplasm (BD‐IPMN) with radiological worrisome features and a pancreatic intraepithelial neoplasia (PanIN) in the context of a chronic IgG4 autoimmune pancreatitis (AIP) is very rare. Here, we report the case of an 81‐year‐old man with symptomatic BD‐IPMN associated with PanIN and IgG4 AIP.

We report a case of symptomatic branch‐duct intraductal papillary mucinous neoplasm (BD‐IPMN) of the pancreas having radiological worrisome features, associated with areas of type 1 and type 2 pancreatic intraepithelial neoplasia (PanIN) in the context of a type 1 autoimmune pancreatitis (AIP).^1^, ^2^, ^3^, ^4^, ^5^

## 2 PRESENTATION OF CASE

In January 2019, an 81‐year‐old Caucasian man was admitted to our surgical department for acute onset of jaundice and abdominal pain. His past medical history revealed hypertension treated with beta blockers; no smoking, alcohol, or drug abuse; weight loss >15 kg in the last three months. No family history of pancreatic neoplasms was reported. At‐home treatments included esomeprazole 40 mg/d and fondaparinux 2.5 mg/d. Physical examination showed normal cardiac conditions (heart rate 77 bpm, blood pressure 125/80 mm Hg, peripheral oxygen saturation 96%) and no abnormal findings in the chest or abdomen. White blood cell count was 11.4 × 10^9^/L with 78.9% neutrophils, and C‐reactive protein (CRP) was 13 mg/L. Liver function tests indicated extrahepatic biliary obstruction with alanine aminotransferase 78 U/L, total bilirubin 8.9 mg/dL, and direct bilirubin 6.4 mg/dL. Blood levels of pancreatic amylase were high (135 U/L) and serum autoimmune antibody tests were positive (IgG4 154 mg/dL). CA 19‐9 was normal (<37 U/mL), whereas CEA was >100 ng/mL (153 U/L).

**Figure 1:**
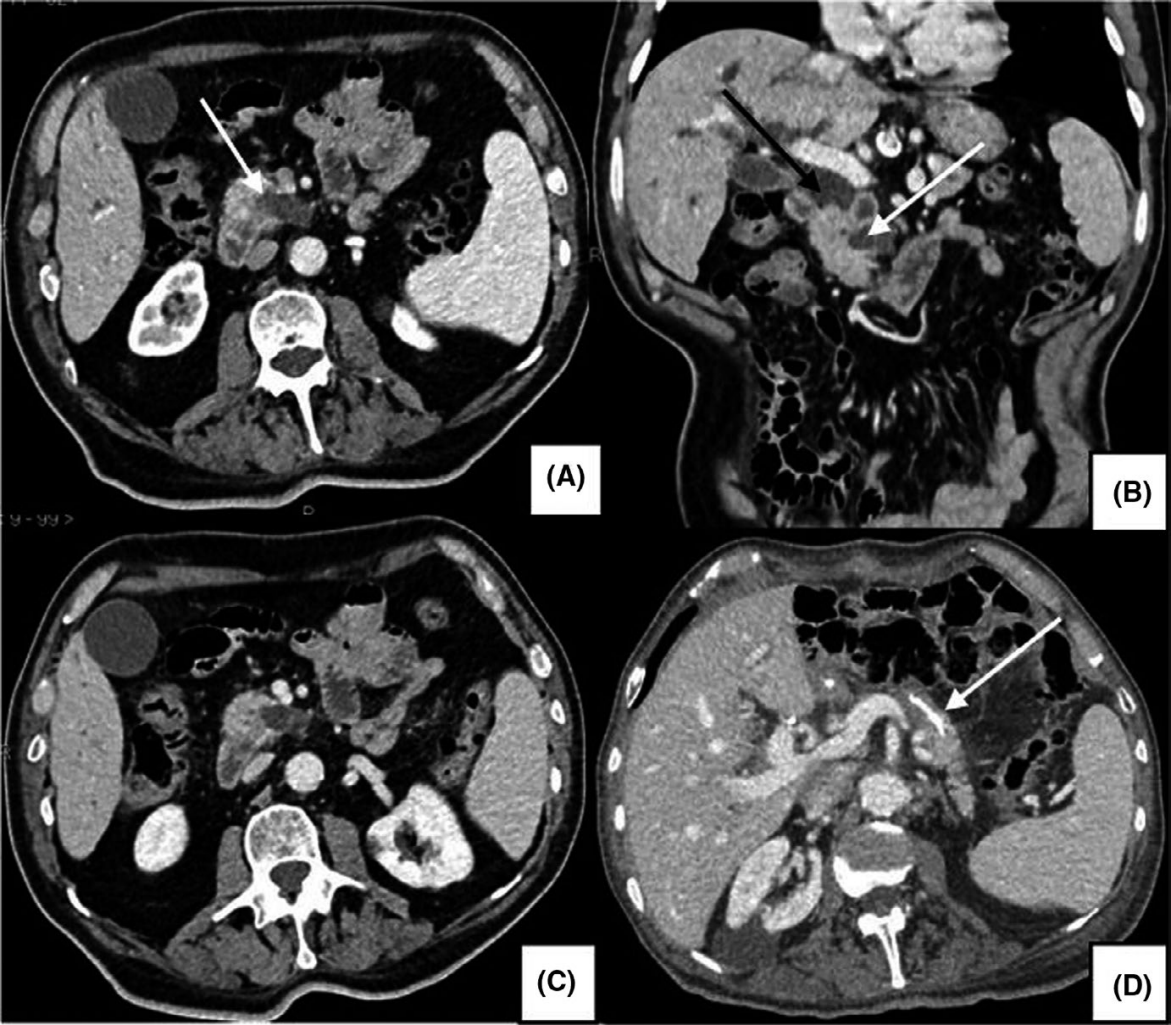
Portal phase CT scans, showing chronic pancreatitis with dilatation of the MPD and parenchymal atrophy (A, B, C). A, axial image: BD‐IPMN with typical communication between the MPD and the cystic lesion (white arrow). B, coronal image: dilatation of the extrahepatic bile duct (black arrow) and upstream dilatation of the MPD (white arrow). D, axial image: pancreatic reconstruction, in which the endo‐Wirsung catheter is visible (white arrow)

Abdominal ultrasonography (US) revealed a 30‐mm hypoechoic lesion in the pancreatic head and uncinate process, causing abrupt interruption and upstream dilatation of the main pancreatic duct (MPD). The lesion was further investigated by contrast‐enhanced computed tomography (CT) and magnetic resonance cholangiopancreatography (MRCP). Both CT and MRI findings supported the diagnosis of pancreatic BD‐IPMN in the context of a chronical pancreatitis (Figure 1): focal cystic lesion of the pancreatic head appearing hyperintense on T1‐weighted and hypointense in T2‐weighted MRI sequences, with multiple internal septations and upstream dilatation of the MPD (10 mm). No liver lesions were detected. CT scans also showed an interstitial pneumonia with infiltrative opacification in the periphery of lungs.

An endoscopic retrograde cholangiopancreatography (ERCP) was performed for jaundice palliation, with stenting of the common bile duct (CBD). Endoscopic ultrasound‐guided fine‐needle aspiration (EUS‐FNA) was carried out to obtain a histological characterization of the pancreatic lesion: the histological examination showed nonspecific cellular atypia (of undetermined significance). Due to the symptoms and the radiological worrisome features of the pancreatic cystic lesion,^6^ the patient underwent pylorus‐preserving pancreaticoduodenectomy (PPPD).

After the resection of the pancreas, the bile duct and the duodenum at 40 mm from the pylorus, restoration of gastrointestinal continuity was performed using the first jejunal loop (Child reconstruction). Firstly, we performed a termino‐lateral pancreaticojejunostomy (PJ) using also a 7 Fr endo‐Wirsung catheter, that was externalized through the Witzel tunneling technique. Secondly, we carried out an end‐to‐side hepaticojejunostomy (HJ) at least 40 mm from the PJ, using a single layer of interrupted, closely spaced, synthetic, nonabsorbable sutures. Finally, we performed a termino‐lateral antecolic isoperistaltic duodenojejunostomy using interrupted, synthetic, absorbable sutures. An intraoperative histopathological examination (frozen section) of the pancreatic and CBD margins was carried out, both to assess the radicality of resection and to exclude the presence of malignancy in the resection margins: no neoplastic abnormalities were found in the examined tissue. The gallbladder was removed. Lymph nodes of the lymphatic stations 8a, 12a, 12p, 12b, 6, 4d, 8p, and 9 were taken with the specimen. Two Penrose‐type surgical drains were positioned: one on the left side to protect pancreatojejunostomy, and the other on the right side to protect hepaticojejunostomy.^6^, ^7^

Nasogastric tube was removed on postoperative day (POD) 1, and clear liquids were allowed on POD 2. Blood glucose was monitored since POD 1, and amylase levels in the drainage liquids were measured on POD 1 and 5. Postoperative course was characterized by a grade A pancreatic fistula (biochemical leak, based on ISGPS 2016 criteria),^8^ without fever, abdominal pain, or leukocytosis. Surgical drainages were removed on POD 5, and the patient was discharged on POD 7. The follow‐up visit on POD 14 confirmed the persistence of good health conditions.

### 2.1 Histopathological analysis

**Figure 2:**
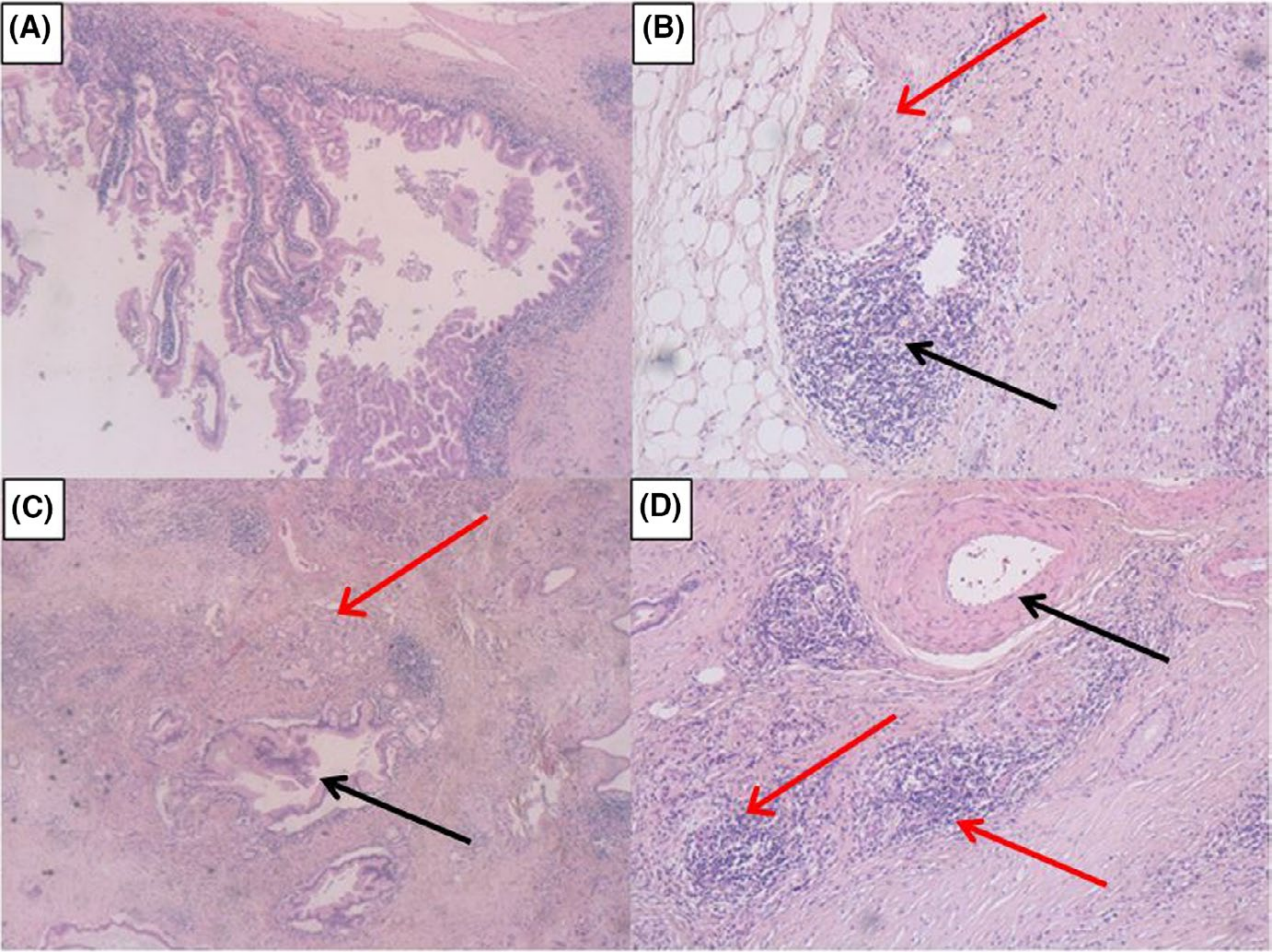
Histopathological analysis. A: the BD‐IPMN (H&E stain, 10X OM). B: an AIP area with perineural (red arrow) and perivascular (black arrow) plasma cell infiltration (H&E stain, 10X OM). C: PanIN type 1 (red arrow) and type 2 (black arrow) lesions (H&E stain, 10X OM). D: clear tropism of inflammatory cells for small pancreatic vessels (red arrow), without infiltration of medium‐sized ones (black arrow) (H&E stain, 10X OM)

Macroscopic and microscopic pathological examination of the pancreatic head revealed the presence of a BD‐IPMN having a main diameter of 30 mm, without intramural nodules or increased wall thickness (Figure 2A). The BD‐IPMN caused upstream dilatation (≥10 mm) of the MPD. The pancreatic parenchyma displayed diffuse autoimmune inflammation (Figure 2B,D) and multiple areas of PanIN, both of type 1 (flat epithelial lesions with tall columnar cells and basally‐located nuclei with abundant supranuclear mucin) and type 2 (papillary mucinous epithelial lesions with nuclear abnormalities, loss of cellular polarity, nuclear crowding, enlarged nuclei, and hyperchromasia) (Figure 2C). Microscopic examination of autoimmune pancreatitis areas showed the characteristic plasma cell infiltrate surrounding small‐sized interlobular pancreatic vessels (Figure 2D) without destruction of the ductal epithelium, associated with swirling fibrosis around ducts and veins. No neoplastic lesions were found on duodenal and periduodenal tissue; lymphovascular invasion was not present; no metastases were detected in the 20 lymph nodes examined (pT1a pN0 based on AJCC 8th Edition staging system). No neoplastic tissue was present in gastric and ileal surgical resection specimens, as well as in the gallbladder.

### 2.2 Immunohistochemistry

**Figure 3:**
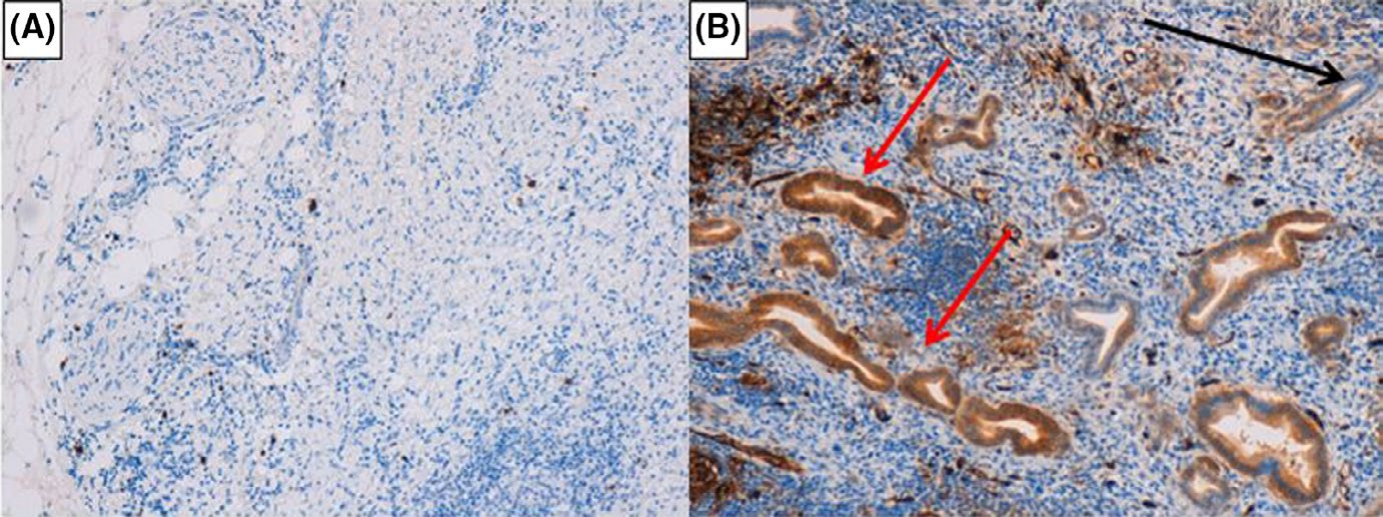
Immunohistochemical analysis. A: high positivity for IgG4 antibodies in the pancreatic parenchyma (IgG4 antibodies IHC, 10X OM). B: fascin‐positive areas of stromal infiltration by the BD‐IPMN (red arrows); the black arrow shows a fascin‐negative area (fascine IHC, 10X OM)

Immunohistochemical (IHC) analysis was performed as described elsewhere.^2^, ^4^ Briefly, using 4 μm formalin‐fixed paraffin‐embedded sections, the analysis was conducted with the standard polymer system and peroxidase methods (antigen retrieval with a heated plate and 0.01 mol/L of citrate buffer, low‐pH, for 40 minutes ‐ Dako Omnis, Denmark A/S). Immunostaining confirmed the presence of abundant IgG4‐positive cells (>10 cells/HPF) in the pancreatic parenchyma (Figure 3A), with diagnosis of IgG4‐related autoimmune pancreatitis. Moreover, fascin staining revealed an invasive component of the BD‐IPMN, with evident fascin‐positive areas of stromal infiltration (DakoCytomation, clone: 55K‐2, 1:100 dilution) (Figure 3B).

## 3 DISCUSSION

In this report, we describe a rare case of pancreatic BD‐IPMN causing jaundice and abdominal pain, which was associated with PanIN and developed on a chronic IgG4 AIP. To our knowledge, this is the first description of such an association in the literature.

AIP is a distinct form of chronic pancreatitis with an autoimmune pathogenesis,^2^ characterized by positive serum autoantibodies and increased serum levels of IgG4. The main clinical features of AIP include obstructive jaundice, recurrent abdominal pain, coexistence of extra‐pancreatic lesions, and a remarkable response to corticosteroids.^5^ Two types of AIP have been identified: type 1 AIP (AIP1),^9^ with diffuse pancreatic fibrosis, the presence of IgG4‐positive cells and a small‐vessel obliterative phlebitis; and type 2 AIP (AIP2),^9^ with characteristic granulocytic epithelial lesions. AIP1 is considered to be the pancreatic manifestation of a systemic disorder called “IgG4‐related disease (IgG4‐RD)”,^10^ in which a variety of systemic lesions can be identified: sclerosing cholangitis (60%), retroperitoneal fibrosis (10%), interstitial pneumonitis (8%, which was present in our patient too), and tubulointerstitial nephritis (8%).^11^ Symptoms, blood test results and clinical images can simulate a pancreatic ductal adenocarcinoma (PDAC, in which IgG4 levels can be slightly elevated too),^12^ a malignant lymphoma,^13^ or other types of pancreatitis. Thus, a careful differential diagnosis is required. A targeted biopsy of the pancreas is the method of choice in cases suspected from a focal form of AIP,^14^ and in our clinic, we prefer it over other diagnostic procedures.

Pancreatic intraepithelial neoplasia (PanIN) can be associated with AIP and can be defined as a preneoplastic lesion in chronic pancreatitis. Some authors consider PanIN as a common precursor of PDAC, even if it can be described as a noninvasive epithelial microscopic neoplasia. PanIN arises from smaller pancreatic ducts and is classified as PanIN‐1, PanIN‐2, and PanIN‐3. PanIN‐1 has characteristic columnar epithelial cells and basally oriented uniform nuclei.^15^ PanIN‐2 lesions are characterized by a high grade of cytoarchitectural complexity, with nuclear changes such as loss of polarity, crowding, size variability, hyperchromasia, and pseudostratification.^15^ PanIN‐3 lesions show the highest grade of dysplasia: papillae and cribriform structures; enlarged, pleomorphic, and nonoriented nuclei^15^, ^16^; mitotic figures and abnormal mitoses. The prevalence of PanIN increases with age.^17^ These lesions are more common in the head of the gland (as it can be observed in PDAC), and PanIN‐3 is more common in PDAC than in chronic pancreatitis. In addition, PanINs are generally more frequent in patients with chronic pancreatitis than in controls. PanIN remains a histologically well‐defined precursor of PDAC: however, the frequency and speed of progression from PanINs to PDAC is an important issue that has not been defined yet.^18^ Particular attention should be paid to the presence of PanIN lesions of any grade in surgical resection margins of a PDAC, even if it doesn't influence patient prognosis and additional surgery is not required.

Pancreatic IPMN is defined as a tumor of the pancreatic duct that produces mucin. Four histologic subtypes of this neoplasm have been described: gastric type, intestinal type, pancreatobiliary type, and oncocytic type.^19^ International guidelines distinguish three different entities based on ductal involvement 20: main‐duct IPMN (MD‐IPMN), which is aggressive and exclusively involves the MPD 21; branch‐duct IPMN (BD‐IPMN), exclusively involving the secondary ducts; and mixed IPMN (M‐IPMN), involving simultaneously the MPD and the branch ducts.^22^ MD‐IPMN and BD‐IPMN have a low/medium malignant potential (up to 20%‐25%), and BD‐IPMN has often a multifocal pattern (involving multiple branch ducts).^23^ The radiological distinction between MD‐, BD‐ and M‐IPMN has prognostic relevance.

The risk of malignancy in pancreatic neoplasms that produce mucin is an important factor that should be properly stratified. Recent guidelines^1^, ^4^, ^22^, ^24^ distinguish tumors with “worrisome features” (WF) or “high‐risk stigmata” (HRS). WF includes cyst size >30 mm, thickened cyst walls, presence of mural nodules, MPD diameter of 5‐9 mm, or an abrupt change in the MPD caliber.^22^ HRS includes enhanced solid component and MPD diameter >10 mm.^22^ In order to obtain a better diagnosis, as well as to avoid risk of a particularly dangerous misdiagnosis, all cysts showing WF should be evaluated by endoscopic US and eventually by a cytological examination (EUS‐FNA). Anyway, if a conservative management approach is chosen, the surveillance should be strict. In case of IPMN with HRS, surgery is mandatory. All patients with cysts without WF should anyway undergo surveillance.^22^, ^24^

In conclusion, in the context of an AIP, the presence of a pancreatic lesion requires histological definition. Delaying surgery may compromise successful tumor resection, due to metastasis development or vascular involvement. In order to rule out the malignant nature of a pancreatic lesion, a percutaneous, or endoscopic biopsy is absolutely mandatory.^24^ This case report seems to confirm the safety of recent guidelines for the surgical management of IPMN.^1^, ^4^, ^24^ In case of symptomatic BD‐IPMN and/or radiological WF, considering that PanIN‐3 lesions are more frequent in the presence of a PDAC, surgical intervention (ie, standard pancreatic resection only) remains mandatory.

## CONFLICT OF INTEREST

The authors have no conflicts of interest to declare.

## AUTHOR CONTRIBUTION

GL: contributed to the conception and design of the study, in the provision of study materials of patients, in the surgical management of patients, in the data analysis and interpretation, in the manuscript writing. LR, AG, BP and ML: contributed in medical oncology patients’ management. GC and FMW: contributed in Histopathological and Immunohistochemical analysis. ADS and VV: contributed in radiological evaluations. MS and FC: contributed in data analysis and interpretation. All authors participated in the collection and/or assembly of data. All authors read, revised and approved the final manuscript.
